# Damage Detection and Localization of Bridge Deck Pavement Based on Deep Learning

**DOI:** 10.3390/s23115138

**Published:** 2023-05-28

**Authors:** Youhao Ni, Jianxiao Mao, Yuguang Fu, Hao Wang, Hai Zong, Kun Luo

**Affiliations:** 1 Key Laboratory of Concrete and Prestressed Concrete Structures of Ministry of Education, Southeast University, Nanjing 210096, China; yhni@seu.edu.cn (Y.N.); kun.luo@foxmail.com (K.L.); 2 School of Civil and Environmental Engineering, Nanyang Technological University, Singapore 639798, Singapore; yuguang.fu@ntu.edu.sg; 3 School of Transportation, Southeast University, Nanjing 210096, China; 230209192@seu.edu.cn; 4 Nanjing Highway Development (Group) Co., Ltd., Nanjing 210096, China

**Keywords:** bridge deck pavement, lane localization of pavement damage, damage detection, lane line semantic segmentation, LaneNet, YOLOv7

## Abstract

Bridge deck pavement damage has a significant effect on the driving safety and long-term durability of bridges. To achieve the damage detection and localization of bridge deck pavement, a three-stage detection method based on the you-only-look-once version 7 (YOLOv7) network and the revised LaneNet was proposed in this study. In stage 1, the Road Damage Dataset 202 (RDD2022) is preprocessed and adopted to train the YOLOv7 model, and five classes of damage were obtained. In stage 2, the LaneNet network was pruned to retain the semantic segmentation part, with the VGG16 network as an encoder to generate lane line binary images. In stage 3, the lane line binary images were post-processed by a proposed image processing algorithm to obtain the lane area. Based on the damage coordinates from stage 1, the final pavement damage classes and lane localization were obtained. The proposed method was compared and analyzed in the RDD2022 dataset, and was applied on the Fourth Nanjing Yangtze River Bridge in China. The results shows that the mean average precision (mAP) of YOLOv7 on the preprocessed RDD2022 dataset reaches 0.663, higher than that of other models in the YOLO series. The accuracy of the lane localization of the revised LaneNet is 0.933, higher than that of instance segmentation, 0.856. Meanwhile, the inference speed of the revised LaneNet is 12.3 frames per second (FPS) on NVIDIA GeForce RTX 3090, higher than that of instance segmentation 6.53 FPS. The proposed method can provide a reference for the maintenance of bridge deck pavement.

## 1. Introduction

Bridge deck pavement is an important part of bridge structure, and the flatness and integrity of the pavement are essential for the traffic function of the bridge [1,2]. The pavement of modern bridges generally consists of asphalt concrete, which belongs to the flexible pavement layer [3]. As the road service life and the traffic volume increases, the damage to the pavement often appears in the form of cracks, ruts, potholes, etc. This damage seriously affects the comfort of drivers, and even affects the safety of driving in the later stages of the damage development [4,5]. Accurate detection and positioning of the damage can provide important management information for the bridge maintenance unit [6]. According to such management information, the unit can timely and accurately deal with the emerging damage to ensure driving safety and smooth traffic.

The traditional damage detection of bridge deck pavement is mainly carried out by vehicle inspection, with engineers taking photos and screening manually [7,8]. The process is laborious and inefficient, and often takes up a large amount of manpower and resources [9]. During the inspection process, it affects the normal traffic operation and safety. Meanwhile, manual visual inspection relies heavily on the experience of inspectors and engineers, which is prone to errors in judgment and has a certain subjectivity [10]. With the continuous development of computer vision, object detection algorithms based on deep learning provide new ideas for damage detection [11]. Object detection algorithms based on deep learning are mainly divided into two-stage algorithms and one-stage algorithms [12]. The two-stage algorithms developed from a selective search to a region proposal network to obtain the region proposals, and predicted the bounding boxes and object scores at each position through the anchor mechanism [13,14]. The Region Convolutional Neural Network (RCNN) and the Faster Region Convolutional Neural Network (Faster-RCNN) are the main representatives. Gou et al. proposed the pavement crack detection method based on the Faster-RCNN, and two branches of the regional proposal network were utilized to realize tasks of classification and position localization. The method has good generalization performance and applicability [15]. Chen et al. introduced densely connected convolution networks and combined a feature pyramid network as the backbone of the Mask Region Convolutional Neural Network (Mask R-CNN) and multi-scale features networks, respectively. The region proposal network was used to generate road damage regions, and the bounding box and area of damage were classified and refined while the mask was created [16]. Sekar et al. proposed an automatic road crack detection and classification method based on a new multi-task Fast Region Convolutional Neural Network (Fast-RCNN) method, and global average pooling (GAP) and region of interest (RoI) alignment techniques were used to detect road cracks [17]. RoI alignment techniques reduce the error in the traditional RoI mapping, and the accuracy and recall of the model on the MIT-CHN-ORR dataset reached 97.9% and 97.3%, respectively.

Although the two-stage algorithms perform well in accuracy, they require large computing power in terms of detection speed. Compared with two-stage algorithms, the one-stage algorithms are generally faster in inference speed with good performances in industrial application [18]. The one-stage algorithms extract the features once to complete the task of object classification and location regression, represented by the Single Shot MultiBox Detector (SSD) and the you-only-look-once (YOLO) series networks [19]. Many one-stage object detection algorithms have been successfully applied to pavement detection. Majidifard classified and quantified pavement damage based on the you-only-look-once version 2 (YOLOv2) network, and developed pavement condition indicators combined with machine learning algorithms [20]. Based on the you-only-look-once version 3 (YOLOv3), Du trained and tested a dataset with 45,788 images from vehicle cameras, and verified that the detection accuracy reached 73.64% under different weather and illumination conditions, and the inference speed was nine times faster than the Faster-RCNN [21]. Ma combined the Prediction-Compensation Generative Adversarial Network (PCGAN) and YOLOv3 to detect and count the cracks in the pavement layer, and introduced an acceleration algorithm and median flow (MF) algorithm to track and observe the cracks [22]. The accuracy of the algorithm was improved to 98.47% under the dataset in this study. Du introduced the Bidirectional Feature Pyramid Network (BIFPN) into the you-only-look-once version 5 (YOLOv5) to improve the multi-scale feature extraction ability, and selected Varifocal Loss to improve the road surface damage detection accuracy [23]. The one-stage algorithms are equivalent to the two-stage algorithms in accuracy and better than the two-stage algorithms in inference speed, but there is still room for improvement. As the latest one-stage algorithm, the state-of-art feature extraction ability of the you-only-look-once version 7 (YOLOv7) makes it faster and more accurate than all known real-time object detection algorithms [24].

Moreover, in terms of pavement damage localization, bridge patrol vehicles are generally equipped with RTK modules, which can accurately determine the location of the vehicle [25]. Furthermore, determining the lane number of pavement damage can accurately locate the damage position, which is beneficial for the bridge management unit to formulate treatment measures [26]. Current research on lane line detection is mainly focused on autonomous or assisted driving. Lane line detection consists of two aspects, traditional image processing technology and deep learning technology [27]. Traditional image processing technology generally includes two aspects, feature-based and model-based lane line detection methods. Feature-based lane line detection methods generally extract features, such as the color [28,29], the shape [30,31], the boundary [32,33] and the texture [34]. Feature-based lane line detection methods require high image resolution and pixel quality. When the scene, especially the pavement background, is complex, the robustness of the algorithm is not high. Model-based lane line detection methods are based on the structured pavement lane distribution form, which is simulated by a geometric model, and the model parameters are obtained by the least square method, the Hough transform and the RANSAC algorithm. Geometric models generally include linear models, parabolic models, hyperbolic models, spline models, etc., as well as combination models [35,36,37,38]. Model-based lane line detection can generally reflect the approximate position and direction of lane lines in the feature extraction stage; nevertheless, the selection of different models in complex scenarios has great impacts on the accuracy of the lane line parameter solution. In addition, the complexity and the number of model parameters increase geometrically in a complex environment, limiting the applicability of such methods.

Aiming at the shortcomings of traditional lane line detection methods, the deep neural network (DNN) has a powerful deep cascade structure [39]. It extracts the hidden features of images through massive neurons, and uses the loss function as the target to update the network weight, so as to complete the lane line detection task in complex scenes. Based on the DNN network, Deeplanes—which directly returns the position of lane lines—was proposed [40]. The network structure is shallow and the accuracy is insufficient. Liu et al. proposed an end-to-end method with the ability to learn the structural information and context information of lane lines, and directly output the parameters of lane line shape [41]. The Spatial Convolutional Neural Network (SCNN) was introduced into lane line detection, which extended the traditional convolution method to the piecewise convolution method in feature layer mapping, and transmitted pixel signals between rows and columns [42]. The above networks are not enough in accuracy improvement, and there still exists a certain gap from the application. Neven et al. proposed an end-to-end LaneNet as a lane line detection model, which is the most widely used model [43]. While performing pixel-level semantic segmentation, the LaneNet combines the vector representation of pixels for embedding clustering, and finally distinguishes lane lines. As a network based on pixel-wise semantic segmentation and instance segmentation, LaneNet greatly improves the semantic segmentation accuracy compared with the previous network. However, the embedding clustering of the instance segmentation part is time-consuming, and the accuracy of the instance segmentation part is not sufficient for application.

To accurately identify the pavement damage to bridges and the lane localization, a three-stage method based on the YOLOv7 and the improved LaneNet is proposed in this study. The main framework of the study is as follows: Firstly, the Road Damage Detection 2022 (RDD2022) asphalt pavement damage dataset is preprocessed and adopted to train the YOLOv7 to form a multi-damage detection model. Secondly, the LaneNet lane line network is pruned with the instance segmentation part removed. Afterwards, an image processing algorithm is proposed to generate an area set of the lane areas. Furthermore, based on the results of the multi-damage detection model and the set of the lane areas, the damage classes and lane localization are determined. Finally, the proposed method is compared and analyzed in the RDD2022 dataset, and is applied to the Fourth Nanjing Yangtze River Bridge in China.

## 2. Proposed Approach

### 2.1. Workflow of Proposed Method

In this section, a three-stage method for damage detection and localization of bridge deck pavement based on deep learning is presented. In this method, the bridge deck patrol vehicle is equipped with a vehicle-mounted camera, and the real-time inference analysis is performed through the on-board server.

**Figure 1:**
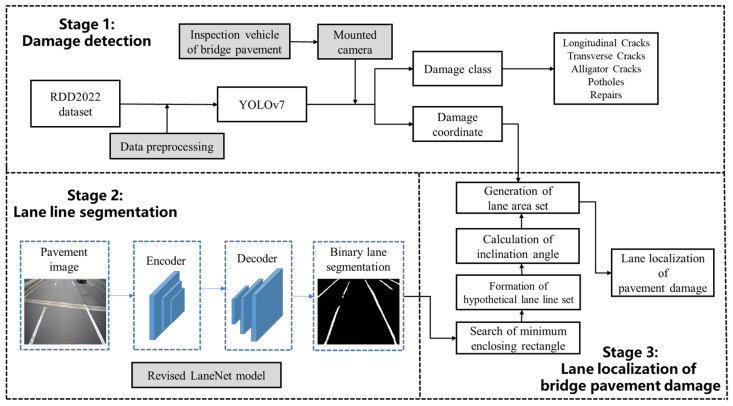
Workflow of proposed damage detection and localization of bridge deck pavement.

Figure 1 shows the workflow of the damage detection and localization of bridge deck pavement. It mainly consists of three stages, including Stage 1 (Damage detection), Stage 2 (Lane line segmentation) and Stage 3 (Lane localization of bridge pavement damage). Stage 1 is the processing of the RDD2022, and the YOLOv7 is trained to obtain a multi-damage detection model. The images obtained by the vehicle-mounted camera are put into the multi-damage detection model to obtain the classes and corresponding pixel coordinates of the pavement damage. In Stage 2, the vehicular-mounted camera images are input into the revised LaneNet semantic segmentation model to obtain the lane line binary images. Stage 3 is a procedure of post-processing binary images of lane lines, and lane numbers are determined based on the order of the lane lines. By comparing the lane area with the damage coordinates of Stage 1, the final damage class and the corresponding lane number are determined.

### 2.2. Stage 1: Damage Detection

#### 2.2.1. YOLOv7 Algorithm

The YOLOv7 is the latest official version of the one-stage object detection network optimized and improved on the YOLOv5 [24]. The network consists of three parts, including backbone, neck and head. The YOLOv7 balances the conflict between parameters, computation and performance. On the Microsoft Common Objects in Context (MS COCO) dataset, the YOLOv7 achieves an average precision (AP) of 56.8% on GPU V100, the highest among real-time detection algorithms, and the frame rate exceeds 30 frames per second (FPS) [24].

**Figure 2:**
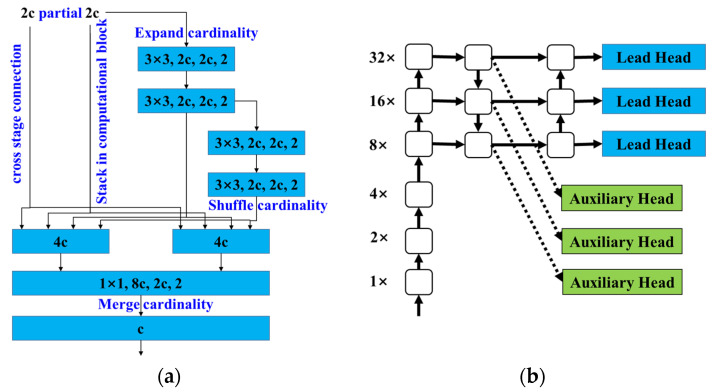
Partial network structure of YOLOv7. (**a**) E-ELAN structure; (**b**) Head with auxiliary head.

Figure 2 is the partial network structure of the YOLOv7. In the backbone network, the extended efficient layer aggregation network (E-ELAN) is proposed. Figure 2a is the E-ELAN structure. In the structure, the cross-stage connection and stack in computational block are used as feature map connection. The E-ELAN maintains the gradient transmission path of the backbone network, and adopts group convolution to promote the cardinality of new features [24]. Methods of shuffle and merge cardinality are utilized to combine the features from different groups. This operation mode can enhance the features learned from different feature maps and promote the usage of calculations and parameters. In each branch operation of the E-ELAN, the input and the output channel dimensions are consistent, which is one of the criteria for efficient networks [44].

The YOLOv7 introduces an auxiliary head on the head side for training. Figure 2b shows the head with auxiliary head [24]. When the YOLOv7 is trained, the auxiliary head participates in training, and the model training would be deeply supervised. The loss of the auxiliary head and the lead head are fused to improve the overall model performance, which is equivalent to a local model ensemble operation at the high level of the network. The YOLOv7 loss function with auxiliary head involved in training is shown in Formulas (1) and (2).

(1) $$L_{\mathrm{Total}}=\sum_{i=1}^{3}L_{C_{i}}\left(M_{\mathrm{Lead}},\lambda_{w}M_{\mathrm{Aux}}\right)$$

(2) $$L_{C}=\left\{L_{\mathrm{obj}},L_{\mathrm{cls}},L_{\mathrm{box}}\right\}$$

where $L_{\mathrm{Total}}$ is the total loss function, $L_{\mathrm{obj}}$ is the confidence loss function, $L_{\mathrm{cls}}$ is the classification loss function, $L_{\mathrm{box}}$ is the localization loss function, $M_{\mathrm{Lead}}$ is the lead head of the model, $M_{\mathrm{Aux}}$ is the auxiliary head of the model, $\lambda_{w}=0.25$ is the weight between $M_{\mathrm{Lead}}$ and $M_{\mathrm{Aux}}$. $L_{C}$ is a collection of loss functions, and $L_{C_{i}}$ is an element of $L_{C}$, where $L_{C_{1}}=L_{\mathrm{obj}}$, $L_{C_{2}}=L_{\mathrm{cls}}$, and $L_{C_{3}}=L_{\mathrm{box}}$.

#### 2.2.2. Pavement Damage Detected by YOLOv7

In Stage 1, the RDD2022 is preprocessed to meet the requirements of bridge pavement detection, and the YOLOv7 is trained with the processed RDD2022 [45]. Afterwards, the pavement images of the bridge deck are obtained by the camera of the bridge patrol vehicle, which are sent into the YOLOv7 multi-damage detection model to obtain the damage classes. These include Longitudinal Cracks, Transverse Cracks, Alligator Cracks, Potholes and Repairs. Finally, the image pixel coordinates of the pavement damage are sent to Stage3 for the lane localization.

### 2.3. Stage 2: Lane Line Segmentation

#### 2.3.1. LaneNet Model

**Figure 3:**
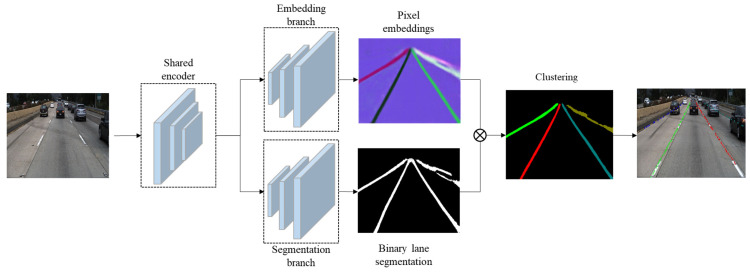
Structure of the LaneNet model.

LaneNet is an end-to-end network proposed by Neven in 2018 [43]. The network adopts instance segmentation to realize the lane line detection task. The pavement image for inspection is used as the input, and the network directly outputs the lane pixels and the corresponding lane line identity (ID) of each pixel. Figure 3 is the structure of the LaneNet model. The LaneNet is similar to traditional semantic segmentation networks, including an encoder network and a decoder network. The decoder contains two branches, the Embedding branch and the Segmentation branch.

The Embedding branch is adopted for the instance segmentation of lane lines. Each pixel of the output feature maps from the decoder corresponds to an n-dimensional vector. In this n-dimensional embedding space, the distance between pixels of the same lane line would be smaller, while that of different lane lines would be larger, so as to distinguish which lane line the pixel belongs to. This branch adopts the one-shot method for distance measurement learning.

The Segmentation branch is to obtain the segmentation results of lane line pixels, and the lane line pixels and non-lane line pixels form a binary image. The open source Tusimple dataset is used for training and validation [46]. In the dataset, lane lines may be blocked by other vehicles, and the ground-truth labeling of lane lines is put through the obstacles, so that the segmentation network can still detect the complete lane line pixels. The cross-entropy loss function is used for the Segmentation branch. As the pixels of the lane line and the background are extremely unbalanced, the bounded-inverse-class-weighting method is adopted to solve the problem of class pixel imbalance [47].

The branch results of the Embedding branch and Segmentation branch are clustered by the Mean-Shift algorithm to obtain the lane line ID result of the instance segmentation. Clustering is part of the post-processing. The Embedding branch provides accurate feature vectors, and these feature vectors can be used to complete the goal of instance segmentation by using clustering algorithms. Missing detection often occurs in instance segmentation, and the lane line clustering process is time-consuming and prone to irreducible noise. Semantic segmentation generally reflects the ground-truth of lane lines, and there exists a phenomenon that a lane line consists of several lane line regions occasionally.

#### 2.3.2. Revised LaneNet Model

**Figure 4:**
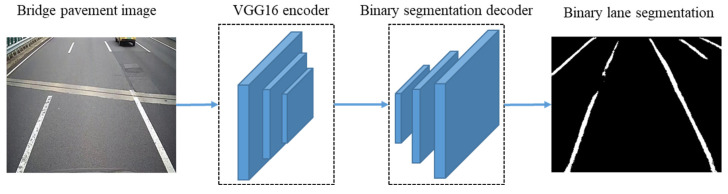
Revised structure of LaneNet model.

The LaneNet network is simplified, and the part of the instance segmentation is removed, including the Embedding branch, Pixel embeddings and Clustering. Figure 4 is the revised structure of the LaneNet model. The VGG16 encoder is adopted as the backbone feature extraction network. The third, fourth and fifth maxpooling layers of the VGG16 encoder output the proposed feature maps. The Binary segmentation decoder is the decoder network that performs two upsampling operations. The feature maps of the third and fourth maxpooling layers of the VGG16 encoder are fused with the two upsampling feature maps of the Binary segmentation decoder, respectively. The binary segment logits with the same size as the image are finally output. The binary segment logits and the binary labels of the mask images are the input to calculate the loss function of the network. The Softmax cross-entropy loss function is adopted as the loss function, as shown in Formulas (3) and (4).

(3) $$L_{\mathrm{Softmax}}=-\frac{1}{N}\sum_{i=1}^{N}\log\frac{e^{W_{C_{i}}^{T}x_{i}+b_{C_{i}}}}{\sum_{j=1}^{\left|C\right|}e^{W_{j}^{T}x_{i}+b_{i}}},i\in \left\{1,2,\ldots ,N\right\}$$

(4) $$C=\left\{C_{1},C_{2},\ldots ,C_{m}\right\},\left|C\right|=2$$

where $L_{\mathrm{Softmax}}$ is the Softmax cross-entropy loss function, $W_{j}$ and $b_{j}$ is the $j^{\mathrm{th}}$ weight and bias of the last fully connected layer of the network, $x_{i}$ a the feed-in neuron of network of the $i^{\mathrm{th}}$ sample, N is the batch size, $W_{C_{i}}^{T}$ and $b_{C_{i}}$ is the target weight and target bias corresponding to goundtruth $C_{i}$, C is the set of real labels in the multi-class problem.

In Stage 2, the vehicular-mounted camera images are input into the VGG16 encoder for feature extraction. Through the Binary segmentation decoder, the binary images of lane line segmentation are output, taken as the input of the post-processing algorithm of Stage 3.

### 2.4. Stage 3: Lane Localization of Bridge Pavement Damage

In Stage 3, the binary lane line segmentation image in Stage 2 is post-processed to achieve the numbered division of the lanes. Thus, an image processing algorithm is proposed to generate an area set of lane areas. The main steps include the search of the minimum enclosing rectangle, the formation of a hypothetical lane line set, the search of minimum enclosing rectangle and the generation of lane area set. Algorithm 1 is the pseudo-code of the proposed image processing algorithm. Given a binary segmentation image $I_{i}$, the task is to generate the set of lane areas $A_{L}{}^{\mathrm{set}}$. In Algorithm 1, PointPolygonTest() is the function of determining whether a point is in a polygon area. By comparing the damage coordinate with $A_{L}{}^{\mathrm{set}}$, the lane localization of bridge pavement damage is achieved.

**Algorithm 1** Pseudo-code of proposed image processing algorithm**Task:** Generate the set of lane areas $A_{L}{}^{\mathrm{set}}$**Input:** area set of binary lane line segmentation $A_{\mathrm{LL}}^{\mathrm{set}}=\left\{a_{\mathrm{ll},i},i=1,2,\ldots ,N^{*}\right\}$, real-time video frame count $F_{v}$, image corner point set $P_{c}=\left\{p_{c,1},p_{c,2}\right\}$, $p_{c,1}$ and $p_{c,2}$ are the lower left corner and the lower right corner of the image, respectively. Final set of lane areas $A_{L}{}^{\mathrm{set}}=\left\{a_{l,i}{}^{\mathrm{set}},i\in M^{*}\right\}$, $M^{*}$ is the number of lane areas, and $N^{*}$ is the number of lane line area.
**Initialization:**

$F_{v}=0,k=1$

While $F_{v}$ > 0Step 1: Traverse $A_{L}$ and search the minimum enclosing rectangle $R_{\min}^{\mathrm{set}}=\left\{r_{i,\min}^{}i\in N^{*}\right\}$ for $A_{L}$Step 2: Sort the coordinates of short sides in the set $R_{\min}^{\mathrm{set}}$ and form the set of coordinate points $P_{c}^{\mathrm{set}}=\left\{p_{i,1}^{e},p_{i,2}^{e},p_{i,3}^{e},p_{i,4}^{e}i\in N^{*}\right\}$Step 3: Sort $P_{c}^{\mathrm{set}}$ by rectange length size and form the hypothetical lane lines set $L_{\mathrm{ll}}^{\mathrm{set}}=\left\{\left[\left(x_{i,1},y_{i,1}\right),\begin{array}{c} \left(x_{i,2},y_{i,2}\right) \end{array}\right],i=1,2,\ldots ,N^{*}\right\}$Step 4: Iterate over $L_{\mathrm{ll}}^{\mathrm{set}}$, when $\left\|l_{\mathrm{ll},i}\right\|>5\left\|l_{\mathrm{ll},i+1}\right\|,\left\{\begin{array}{c} l_{\mathrm{ll},i} \end{array}=\left[\left(x_{i,1},y_{i,1}\right),\begin{array}{c} \left(x_{i,2},y_{i,2}\right) \end{array}\right]i\in N^{*}\right\}$, delete $\left\{\begin{array}{c} l_{\mathrm{ll},j} \end{array}=\left[\left(x_{j,1},y_{j,1}\right),\begin{array}{c} \left(x_{j,2},y_{j,2}\right) \end{array}\right]j=i+1,i+2,\ldots ,N^{*}\right\}$ and form the set of new lane lines $L_{\mathrm{ll}}^{\mathrm{set},1}=\left\{\left[\left(x_{i,1},y_{i,1}\right),\begin{array}{c} \left(x_{i,2},y_{i,2}\right) \end{array}\right],i=1,2,\ldots ,N^{*}-j+1\right\}$Step 5: Based on $L_{\mathrm{ll}}^{\mathrm{set},1}$, calculate the set of inclination angle $\theta_{\mathrm{ll}}^{\mathrm{set},n}=\left\{\theta_{i},i=1,2,\ldots ,N^{*}-j+1\right\}$ Step 6: Traverse $\theta_{\mathrm{ll}}^{\mathrm{set},n}$, when $\left\|\theta_{k}-\theta_{k+1}\right\|<5^{0},\left\{k=1,2,\ldots ,N^{*}-j\right\}$, merge $\begin{array}{c} l_{\mathrm{ll},k} \end{array}$ and $\begin{array}{c} l_{\mathrm{ll},k+1} \end{array}$, and form the final set of lane line collection $L_{\mathrm{ll}}^{\mathrm{set},2}=\left\{l_{\mathrm{ll},i}^{\mathrm{set}},i=1,2,\ldots ,N^{*}-j+1\right\}$Step 7: Iterate over $L_{\mathrm{ll}}^{\mathrm{set},2}$, when $\mathrm{PointPolygonTest}\left(p_{c,j},l_{\mathrm{ll},i}^{\mathrm{set}},l_{\mathrm{ll},i+1}^{\mathrm{set}}\right)>0$, form $a_{l,i}{}^{\mathrm{set}}=\left(I_{c,j},l_{\mathrm{ll},i}^{\mathrm{set}},l_{\mathrm{ll},i+1}^{\mathrm{set}}\right)$Step 8: Generate the final lane area set $A_{L}{}^{\mathrm{set}}=\left\{a_{l,i}{}^{\mathrm{set}},i\in M^{*}\right\}$Step 9: $F_{v}=F_{v}+1,$ repeat Step 1 to Step 8End**Output:** $A_{L}{}^{\mathrm{set}}$

## 3. Training and Comparison of Damage Detection Model

### 3.1. Dataset Preparation

The RDD2022 dataset adopted in this study is from the Crowdsensing-based Road Damage Detection Challenge. The Conference is a part of the IEEE International Conference on Big Data 2022 [45]. The RDD2022 dataset is collected by cars, motorbikes and drones from six countries, including Japan, India, the Czech Republic, Norway, the United States and China. The annotated dataset contains six classes, of which four are major damage types. The main damage classes are Longitudinal Cracks, Transverse Cracks, Alligator Cracks, Potholes, and other classes are Repairs and Manhole covers.

**Figure 5:**
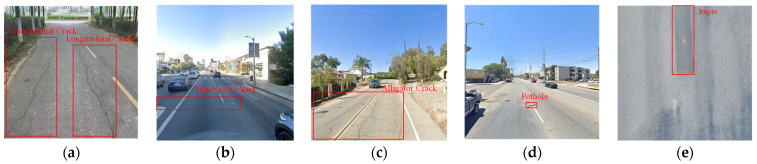
Damage classes and labeling of RDD2022 dataset. (**a**) Longitudinal Crack; (**b**) Transverse Crack; (**c**) Alligator Crack; (**d**) Pothole; (**e**) Repair.

According to the real needs of bridge deck inspection, five classes were selected for statistical analysis. Figure 5 shows the damage classes and labeling of the RDD2022 dataset. Longitudinal Cracks, Transverse Cracks, Alligator Cracks, Potholes are damage images, and the Repair needs rechecking by the maintenance unit.

**Figure 6:**
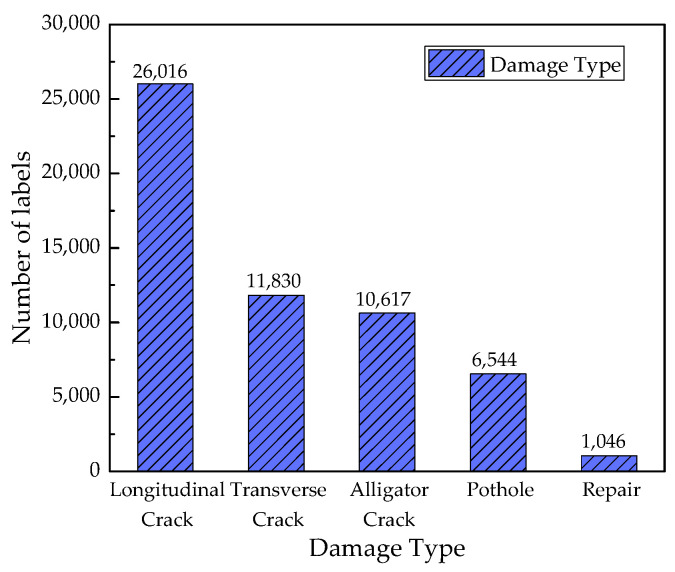
Distribution of damage labels in the RDD2022 dataset.

The RDD2022 includes 47,420 asphalt pavement images, of which the number and distribution of the five classes are shown in Figure 6. The maximum number of Longitudinal crack-labeled tags is 26,016, and the minimum number of Repair-labeled tags is 1046.

### 3.2. Model Training of YOLOv7

YOLOv7 training was performed on a server equipped with NVIDIA GeForce RTX 3090 with 24GB of video memory. The initial learning rate was set to 0.01, and the Stochastic Gradient Descent (SGD) algorithm with momentum was adopted, and the momentum was set to 0.937. The weight decay was set to 0.0005. Batch size was set to 128 and the number of epochs was set to 300. For the dataset with an average quality of labeled data, the weight parameter for the true value in the loss function was set to the scale from 0 to 1, that is, the label-smoothing was set to 0.95. Transfer training was adopted, and the preloading weights were based on the training results of the MS COCO dataset.

**Figure 7:**
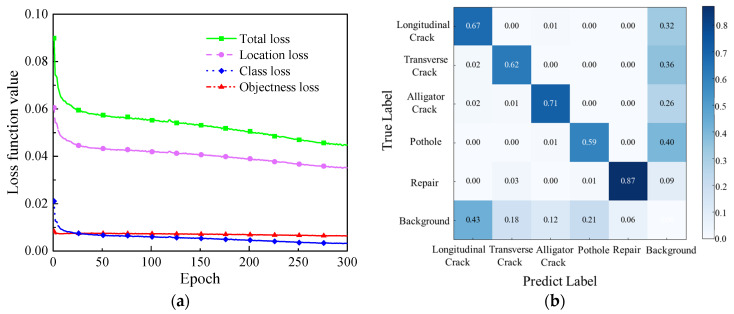
YOLOv7 training results. (**a**) The curve of loss function value; (**b**) Confusion matrix diagram.

Figure 7 shows the YOLOv7 training results. Figure 7a shows the curve of loss function value. Total loss includes location loss, class loss and objectness loss. The loss function value decreases rapidly before 50 epochs, among which class loss decreases the fastest. The value of the loss function decreases steadily and then converges. Figure 7b is the confusion matrix diagram of the YOLOv7 model. The probability of a Pothole being incorrectly identified as background is 0.40, which is the highest. It can be seen that a Pothole is easier to be confused with the background due to its irregular shape. Due to the obvious features and relatively large scale, Repair has the highest detection accuracy of 0.87.

### 3.3. Algorithm Performance Analysis

**Figure 8:**
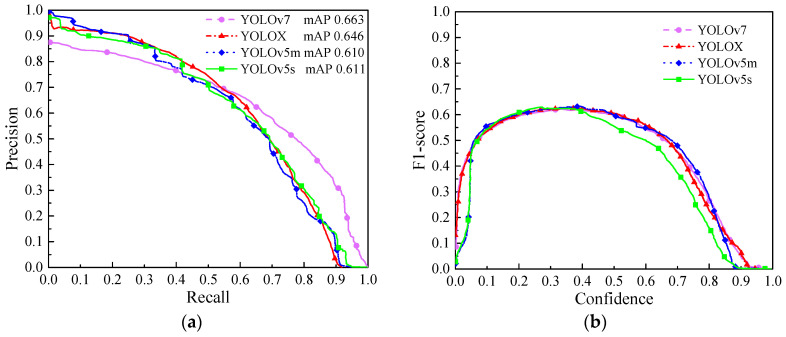
Comparative analysis of YOLO series algorithms. (**a**) Precision-recall curve; (**b**) F1-score-confidence curve.

Figure 8 shows the comparative analysis of the YOLO series algorithms. Figure 8a is the precise-recall (P-R) curve of the YOLO series. In general, the recall decreases as the precision increases. When the P-R curve of the two models intersects, the quality of the model is judged by the area under the curve, and the model with the larger area is better. As shown in Figure 8a, when the recall rate is less than 0.5, the precision of YOLOv7 is slightly lower than that of YOLOX and YOLOv5. When the recall rate is between 0.9 and 1.0, the precision of YOLOv7 is much larger than that of the other models. Meanwhile, the mean precision of the other models was below 0.1. Mean average precision (mAP) is the area enclosed by the P-R curve and the horizontal axis. In general, YOLOv7 has a highest area and balanced curve, with a mAP value of 0.663.

Figure 8b is the F1-score-confidence curve. The curve has the characteristics of three segments, namely, the ascending segment, the stationary segment and the descending segment. In the ascending segment, YOLOv7 has a much higher F1-score than the other models at the same confidence level. In the stationary segment, the model trends are consistent, and YOLOv7 is close to YOLOv5m, and higher than the other models. In the descending segment, YOLOv7 has intersections with other model curves and is higher than the other models. Generally, the confidence threshold of object detection for pavement damage is 0.5; thus, the YOLOv7 model is superior to other models in terms of accuracy and recall rate, which is consistent with the conclusions of the P-R curve.

### 3.4. Comparison of Damage Detection Results

For the conciseness of the detection results, the damage classes were linked separately according to simple label names, as shown in Table 1. There are five concise label names, including D00, D10, D20, D40 and Repair. The confidence threshold was set as 0.5, and the intersection over union (IOU) threshold was set as 0.45.

**Figure 9:**
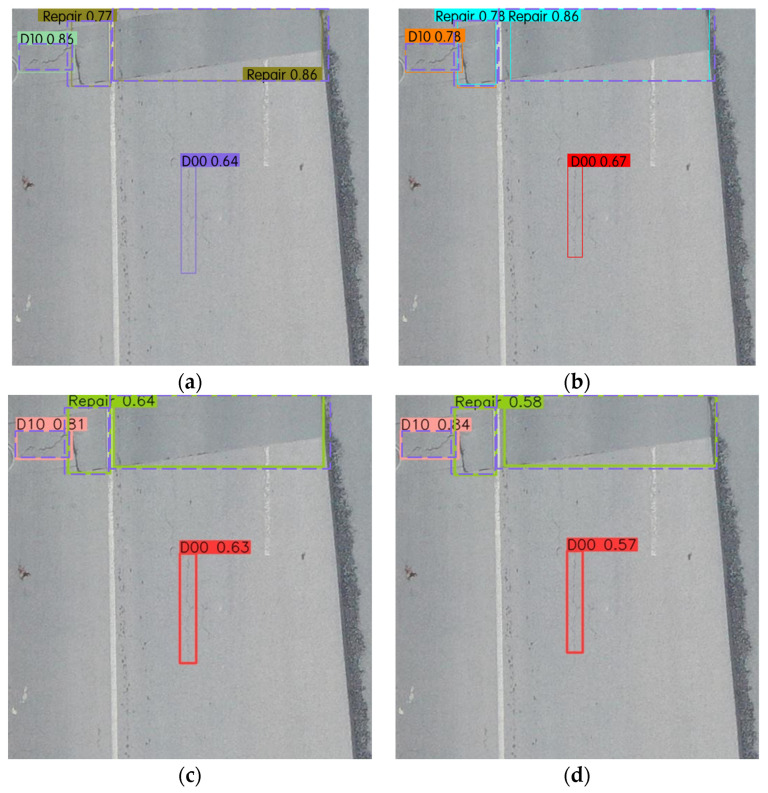
Examples of model detecting results comparison. (**a**) Detection result of YOLOv7; (**b**) Detection result of YOLOX; (**c**) Detection result of YOLOv5m; (**d**) Detection result of YOLOv5s.

The detection results of the YOLO series network were compared by visual inspection. Figure 9 shows examples of the model detecting results comparison. Figure 9a–d show the detection results of YOLOv7, YOLOX, YOLOv5m and YOLOv5s, respectively. The purple dashed line in the figure is the ground-truth bounding box of the dataset label, and the remaining lines are the prediction boxes. Each prediction box was matched with a prediction label, indicating the class of damage being predicted and the prediction confidence (probability from 0 to 1). In general, the YOLOv7 prediction box had the highest IOU value with the ground-truth bounding box, followed by YOLOv5m. YOLOX and YOLOv5s have some discrepancy with the ground-truth bounding boxes, respectively. D10 is Transverse cracks, and YOLOv7 has the highest prediction confidence, reaching 0.86. In terms of Repairs prediction confidence, YOLOX is about the same as YOLOv7, and higher than the other two models. It is worth noting that the Longitudinal Crack (the D00 label) is not annotated. All four models correctly detected the D00 damage.

**Figure 10:**
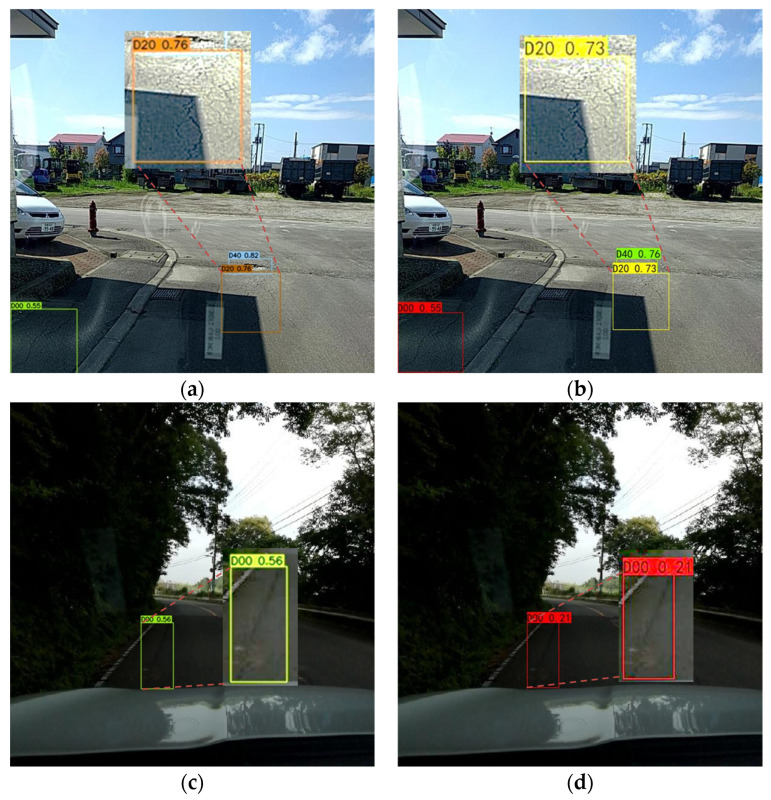
Model detection results under dark light conditions. (**a**) Under partial shadow occlusion conditions by YOLOv7. (**b**) Under partial shadow occlusion conditions by YOLOX. (**c**) Under full shadow occlusion conditions by YOLOv7. (**d**) Under full shadow occlusion conditions by YOLOX.

Figure 10 shows the model detection results under dark light conditions. Figure 10a,b show the damage detection under partial shadow occlusion conditions by YOLOv7 and YOLOX, respectively. It can be found that the D20 damage is Alligator Cracks, partially located in the shadow, and partially in the sun. Human eye cannot accurately identify the Alligator Cracks under the shadow, and it is easy to miss in the labeling process [45]. However, YOLOv7 and YOLOX network models correctly identified D20 damage. The bounding box content was enlarged by 200%, the brightness of the image was increased by 50%, and the Alligator Cracks under the shadow can be clearly seen. Compared with the YOLOX model, YOLOv7 had a higher detection confidence of 0.76. Figure 10c,d show the damage detection under full shadow occlusion conditions by YOLOv7 and YOLOX, respectively. It can be found that the D00 damage is Longitudinal Cracks, which are totally in the shadow. YOLOv7 detected D00 with a confidence of 0.56. However, YOLOX only had a confidence of 0.21, and it was not considered to have correctly identified D00 according to the confidence threshold of 0.5. The detection accuracy of YOLOv7 is higher than that of human eyes and that of the YOLOX network under poor lighting conditions.

## 4. Lane Localization of Pavement Damage

### 4.1. Lane Line Segmentation Effect

**Figure 11:**
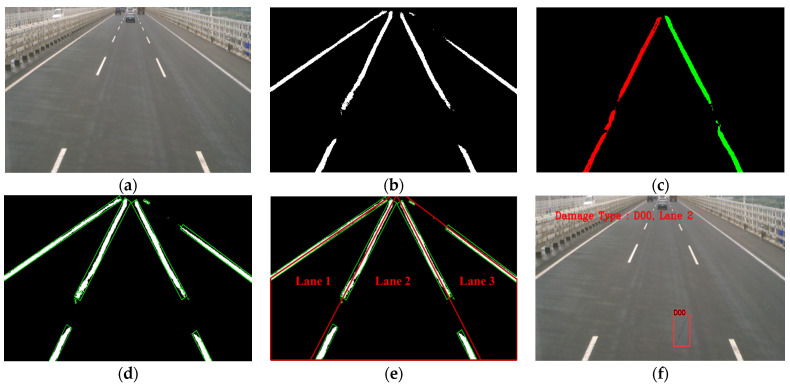
Process diagram of lane localization. (**a**) Original image; (**b**) Semantic segmentation; (**c**) Instance segmentation; (**d**) Minimum enclosing rectangle; (**e**) Lane ordering by post-processing; (**f**) Lane localization of the damage.

Figure 11 shows the process diagram of lane localization. Figure 11a shows the original image of the bridge pavement, where there are three lanes. Figure 11b is the binary image of the lane line semantic segmentation. In this process, the lane lines are segmented out through the revised segmentation network. In this study, the traditional VGG16 is used as the backbone feature network.

Figure 11c shows the instance segmentation result of the lane lines. It can be seen that the first lane line and the fourth lane line in this image are missed. The reason is that the embedding vector learning ability of the lane line is not sufficient enough in the clustering process, and the one-hot encoding of the two lane lines is not successful. Figure 11d shows the minimum enclosing rectangle of the lane lines. Some lane dashed lines are partially fully connected.

Figure 11e shows lane ordering by the post-processing algorithm. It can be seen that the thick red line is the lane line obtained by the post-processing algorithm. The small area lane lines were removed as the outlier noise, while the lane lines of larger areas were involved in the fitting of the main area lane lines. In the process of vehicle inspection, the lower left and the lower right corner of the image are always the lane areas. Thus, the two corners are taken into account to determine the relationship between the two corners and the lane. As shown in the figure, the lower left corner point is located in the range of the first lane, and the lower right corner point is located in the range of the third lane. Therefore, the first corner point is connected with the first lane line and the second lane line to form the first lane area. It is the same with lane 2 and lane 3. Thus, a complete division of the lane area is formed.

Figure 11f shows the lane localization of the damage. The damage detection model of YOLOv7 was used to detect the images, and the damage classes and pixel coordinates were obtained. The pixel coordinates were compared one by one with the lane area of Figure 11e, where the damage was a Longitudinal crack, labeled D00, and located in the second lane.

### 4.2. Localization Accuracy Analysis

Data on bridge pavement (including highways and national roads) with normal traffic were selected for testing. There is a middle belt on the pavement, and passing vehicles consist of large trucks and other automobiles. There exists a phenomenon that vehicles often occupy lane lines, including the solid line and the dashed line.

**Figure 12:**
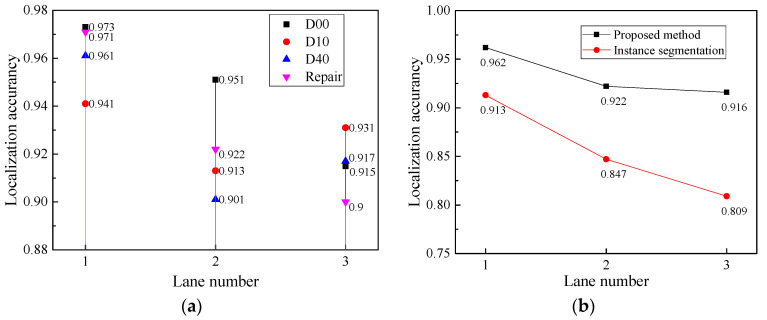
Comparison of lane localization accuracy. (**a**) Lane localization accuracy of each class. (**b**) Comparison between proposed method with instance segmentation.

Figure 12 shows the comparison of lane localization accuracy. Figure 12a shows the lane localization accuracy of each class. This study calculates the localization accuracy of each lane for four different types of damage, including D00, D10, D40 and Repairs. In general, as the number of lanes increases, the lane localization accuracies of all types of damage decline. The reason is that the detection of subsequent lane numbers depends on the preceding lane. Furthermore, all localization accuracies are above 0.90. D00 Longitudinal Cracks has the highest localization accuracy of 0.973 in the first lane.

Figure 12b is the comparison between the proposed method with instance segmentation. Generally, the accuracy of the proposed method is larger than that of the instance segmentation method in each lane. The average accuracy of the proposed method in three lanes is 0.962, 0.922 and 0.916, respectively. The mean average accuracy of this method is 0.933, and the mean average accuracy of the instance segmentation method is 0.856, an increase of 8.9%.

**Figure 13:**
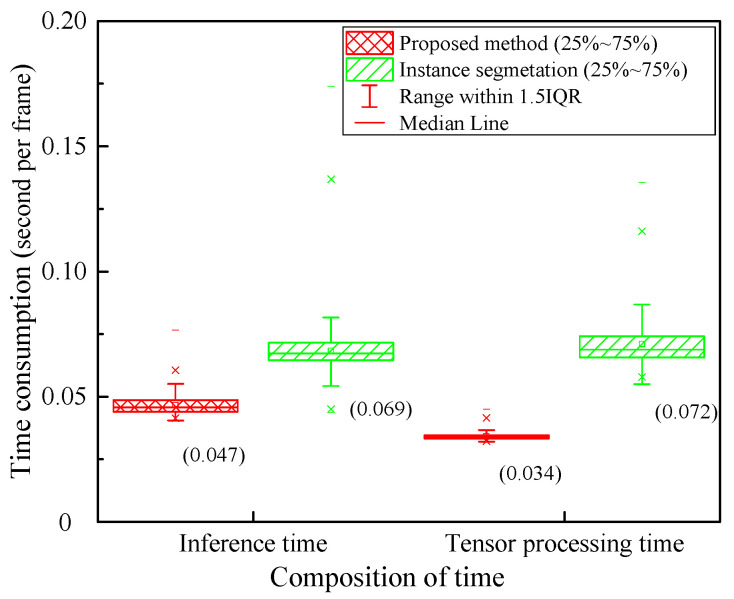
Box plot of algorithm computing costs.

Figure 13 is the box plot of algorithm computing costs. The algorithm computing costs include two parts, inference time and tensor processing time. The interquartile range (IQR) of inference time and tensor processing time of the proposed method is smaller than that of the instance segmentation. The range within 1.5 IQR of the inference time and tensor processing time of the proposed method is also smaller than that of the instance segmentation. The inference time of the proposed method is 0.047 s per frame, and the tensor processing time is 0.034 s per frame. The inference speed of the proposed method is 12.3 FPS, which is almost twice that of the instance segmentation (6.53 FPS).

### 4.3. Engineering Application

In order to verify the applicability of the proposed method, the Fourth Nanjing Yangtze River Bridge was taken as an example to detect and locate the deck pavement damage. The bridge is the largest cross-border suspension bridge in China, with two towers and three spans and a two-way six-lane highway. It has been open to traffic for more than 10 years since 2012. There exists some damage in the deck pavement, and which requires regular inspection and repair.

Table 2 shows the frequency of the pavement damage occurrence over the years. The main damage from 2015 to 2021 included Scratch, Indentation, Construction joint and other cracks. Scratches on the pavement surface are generally caused by the sharp parts of the accident vehicle acting on the pavement layer, and their shape is like cracks, so they are classified as cracks. Indentations are mainly caused by the local force of the pavement being greater than its compressive strength, showing a concave shape; these are classified as Potholes. As a routine inspection item, Construction joint is classified as crack. It is seen from Table 2 that scratches are the main type of damage to the pavement in the period of 2019 to 2021; they may be caused by the increased accident rate resulting from the heavier traffic.

The damage images of the bridge pavement are generally a local view, and the lanes cannot be completely presented. Therefore, the vehicle inspection images of the bridge were combined with the original damage images to form the synthetic damage images. The proposed three-stage method was adopted to apply in the inspection project.

**Figure 14:**
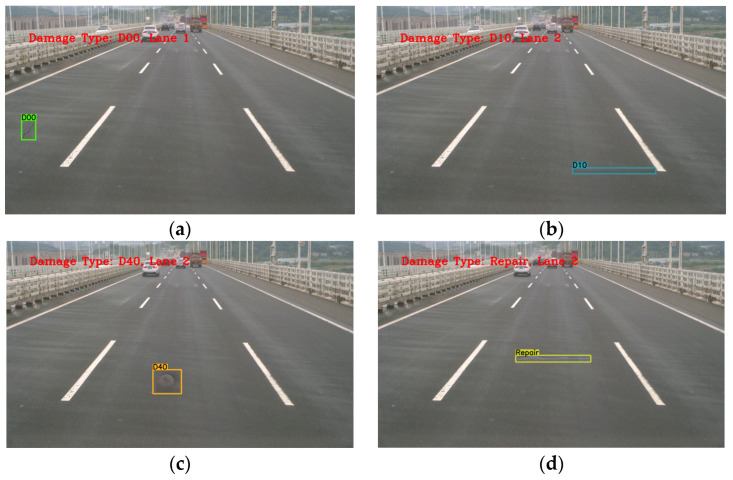
Examples of damage detection and lane localization. (**a**) Longitudinal cracks; (**b**) Transverse cracks; (**c**) Potholes; (**d**) Repairs.

Figure 14 shows the examples of damage detection and lane localization. Figure 14a shows a Longitudinal Crack, which is the scratch. The detection class is D00, and the damage is located in the first lane. Figure 14b shows Transverse cracks. The detection class is D10, and the damage is located in the second lane. Figure 14c shows a Pothole, which is the indentation of pavement. The detection class is D40, and the damage is located in the second lane. Figure 14d shows the asphalt repair of pavement located in the second lane, and the detection class is Repairs.

## 5. Conclusions

In this study, a three-stage method based on YOLOv7 and the revised LaneNet for bridge pavement damage detection and lane localization was proposed. Firstly, the RDD2022 asphalt pavement damage dataset was preprocessed to train YOLOv7, and a multi-damage detection model was formed. The damage includes Longitudinal cracks, Transverse cracks, Alligator cracks, Potholes and Repairs. Secondly, the LaneNet network was pruned to retain the semantic segmentation part, and VGG16 was adopted as the encoder. The binary image of the lane line was obtained from the revised LaneNet model. Finally, an image processing algorithm was proposed to generate an area set of lane areas. By comparing the damage coordinates with the lane area, the final damage class and lane localization of bridge deck pavement were obtained. The proposed method was compared and analyzed in the RDD2022 dataset, and was applied on the Fourth Nanjing Yangtze River Bridge. The following conclusions were obtained by this method:

(1) At the confidence threshold of 0.5 and the IOU threshold of 0.45, the mAP value of YOLOv7 in the preprocessed RDD2022 dataset reaches 0.663, higher than other models in the YOLO series;
(2) The lane localization accuracy of the revised LaneNet is 0.933, higher than that of instance segmentation. Operated on NVIDIA GeForce RTX 3090, the inference speed of the revised LaneNet is 12.3 FPS, almost twice that of the instance segmentation;
(3) The proposed method was verified on the RDD2022 dataset and applied to the fourth bridge of the Yangtze River in Nanjing, providing a reference for the maintenance of bridge deck pavement.

## Figures and Tables

**Table 1:** Labels corresponding to damage type.

Label Name	Damage Type
D00	Longitudinal Cracks
D10	Transverse Cracks
D20	Alligator Cracks
D40	Potholes
Repair	Repairs

**Table 2:** Frequency of the pavement damage occurrence over the years.

Damage Type	Direction	Year						
		2015	2016	2017	2018	2019	2020	2021
D00, D10 (Scratch)	upriver	3	9	14	6	17	17	13
	downriver	2	3	1	3	13	12	3
	In total	5	12	15	9	30	29	16
D40 (Indentation)	upriver	2	0	0	0	0	1	3
	downriver	0	0	0	0	0	1	1
	In total	2	0	0	0	0	2	4
D10 (Construction joint)	upriver	1	1	0	0	0	0	0
	downriver	1	1	0	0	0	0	0
	In total	2	2	0	0	0	0	0
Repairs (Other cracks)	upriver	0	0	0	0	1	3	2
	downriver	0	0	0	0	1	1	6
	In total	0	0	0	0	2	4	8

## Data Availability

Data will be made available on request.
